# Renewals

**Published:** 1883-11-20

**Authors:** 


### *RENEWALS.*

We shall expect our subscribers all to renew their subscriptions for the next year, and, unless specially directed to the contrary, will enter all upon the list for 1884. From all points we are complimented upon the practical character of our Journal—and there are hundreds who have persistently stuck to the RECORD during its entire existence of thirteen years, and who have uniformly testified to its superior practical value and usefulness to the practitioner.

